# Occurrence and severity of myasthenic crisis in an unselected Turkish cohort of patients with myasthenia gravis

**DOI:** 10.3389/fneur.2023.1201451

**Published:** 2023-07-13

**Authors:** Selen Ozyurt Kose, Ezgi Nazli, Kemal Tutkavul, Nils Erik Gilhus

**Affiliations:** ^1^Department of Clinical Neurophysiology, Marmara University Pendik Teaching and Research Hospital, Istanbul, Turkey; ^2^Department of Neurology, Health Sciences University Haydarpasa Numune Teaching and Research Hospital, Istanbul, Turkey; ^3^Department of Neurology, Haukeland University Hospital, Bergen, Norway; ^4^Department of Clinical Medicine, University of Bergen, Bergen, Norway

**Keywords:** myasthenia gravis, myasthenic crisis, ventilation support, prognosis, treatment

## Abstract

Myasthenia gravis (MG) is a disorder of the neuromuscular junction that can deteriorate into myasthenic crisis, involving weakness of bulbar and respiratory muscles. In this study, we describe the clinical manifestations of myasthenic crisis, identify risk factors, and examine treatments and outcomes. All 95 patients with generalized MG treated at our center during the last 10 years were included in this retrospective study. We collected data from the patients' records, including clinical follow-ups, muscle antibodies, thymic status, and treatments. The characteristics of patients who did and did not experience myasthenic crisis were compared. Features of all myasthenic crises were also assessed. Twelve patients (13%) developed myasthenic crisis during the observation period. Men were more often affected at older ages. Seven patients experienced multiple myasthenic crises. Thymoma increased the risk of a crisis, whereas thymic hyperplasia decreased the risk. Myasthenic crises were more common in the summer months. No patients died during a myasthenic crisis. Risk factors for myasthenic crisis were thymoma, older age, MuSK antibodies, and previous crises. Individualized and active immunosuppressive treatment and optimal intensive care during crises provide a good outcome for patients with generalized MG.

## Introduction

Myasthenia gravis (MG) is caused by structural and functional changes in the postsynaptic membrane induced by autoantibodies against the acetylcholine receptor (AChR) or muscle-specific kinase (MuSK) (1). This results in impaired neuromuscular transmission and fluctuating weakness in specific muscle groups, preferentially in the ocular, bulbar, and upper extremity muscles. MG is categorized into subgroups with respect to the presence of thymic pathology, autoantibody type, early vs. late onset (before or after the age of 50 years), and the muscle groups that are affected (2, 3).

MG tends to be more severe in elderly patients, in those with a thymoma, and in non-thymectomized early-onset patients (2, 4). Active drug treatment and early thymectomy benefit MG patients. MG related mortality has declined, but nationwide database analyses still show an increased mortality rate in patients with MG as compared to the general population, hthis rate is also slightly higher in male patients. The MG mortality rate is currently reported to be ~1.76–1.41 (5–8). The main risk factors for in-hospital deaths are older age and respiratory failure (9).

Myasthenic crisis, involving respiratory muscle weakness and a need for respiratory support, represents the most severe manifestation of the MG clinical spectrum. In previous years, this form of crisis was found to be experienced at least once by 15%−20% of MG patients (10), but it appears to occur less frequently in recent studies. In a multicenter study including 59,243 patients, 1,468 patients (2.5%) were hospitalized with myasthenic crisis between 2016 and 2018 (11). Moreover, myasthenic crisis mortality has decreased (12–14). Patients with respiratory weakness and symptoms that indicate a crisis should be hospitalized immediately and observed continuously for respiratory function. With early recognition and active emergency treatment, intensive care unit (ICU) hospitalization and ventilatory support may be avoided. First-line treatment options for myasthenic crisis and severe exacerbations are plasma exchange or IVIG administration (2, 3, 15). New therapies, such as anti-complement and anti-neonatal FcRn drugs, are effective in MG patients and are currently being investigated for treatment of myasthenic crisis (16).

We conducted a single-center retrospective study of an unselected Turkish MG cohort to investigate the occurrence and nature of exacerbations and crises, with the aim of identifying pre-crisis MG features and risk factors, determining key clinical parameters during deterioration, and assessing the outcomes of treatment.

## Materials and methods

We identified all patients with a diagnosis of MG (ICD-10: G70.0) who were treated at the Haydarpasa Numune Training and Research Hospital (HNH) from 2011 to 2021. Patients who had only ocular symptoms and signs were excluded (28 patients). We also excluded those with Lambert–Eaton myasthenic syndrome (three patients). Our sample thus consisted of 95 patients with generalized MG. All included patients were over the age of 18 years and were followed for at least 1 year at the HNH neuromuscular diseases outpatient clinic. This was a retrospective study, and MGFA or MG-ADL scores had not been noted in most patient files. We were therefore unable to include such scores in our analyses.

The HNH Scientific Ethical Committee approved this study with reference number HNEAH-KAEK 2022/KK/150.

The MG patients were grouped according to whether or not they had experienced a myasthenic crisis during the last 10 years. We applied the formal 2016 international consensus-based guidance on myasthenic crisis, defining this as an aggravation of bulbar or generalized muscle weakness with deterioration on the muscle strength scale, plus respiratory failure as a result of muscle weakness, requiring non-invasive ventilatory support or mechanical ventilation (3). Two episodes that occurred in MG patients where a cardiopulmonary disorder was the main reason for respiratory insufficiency were excluded. Five episodes occurring immediately post-thymectomy were also excluded.

All patients underwent chest CT scan for thymus examination. For patients who underwent a thymectomy, diagnoses of thymoma and thymic hyperplasia were made according to histopathological examination. We collected venous blood for respiratory function analysis at the time of each episode of myasthenic crisis. Partial pressure carbon dioxide (pCO_2_), partial pressure oxygen (pO_2_), and pH values were examined. However, the results of arterial blood measurements were not permanently stored in our hospital recording system. We also measured single breath counts before the need for ventilatory support. Arterial blood gas test results were not available for most patients.

In descriptive analyses in terms of demographics and clinical characteristics, categorical data are presented in the form of frequencies (*n*) and percentages (%), while continuous data are presented in the form of mean values with standard deviations. Categorical variables were compared between groups using the chi-square test, the chi-square test with Yates' correction, and Fisher's exact test (the latter in cases where numbers were below five). Continuous variables were compared using independent *t*-tests. Analyses were performed using GraphPad Prism 9.0 (GraphPad Software, Inc., San Diego, CA), and the threshold for significance was defined as *p* < 0.05.

We assessed the clinical features of MG, relevant comorbidities, laboratory test results, interventions during myasthenic crisis, and treatment escalations. Predictors of hospital stay length (age, gender, thymoma, pCO_2_ value, comorbidities, decrease in single breath count, and thymectomy) were analyzed using a partial least squares (PLS) regression model due to the small sample size and non-normally distributed data. pCO_2_ was chosen as a reflection of the blood tests due to the correlation between pO_2_ and pCO_2_ values. The assumption of independence of the explanatory variables was tested by considering the variance inflation factor (VIF).

## Results

The sample consisted of 53 women and 42 men with a diagnosis of generalized MG, who were followed from 2011 to 2021. Their average disease duration was 10.2 years. The mean age was 52.6 ± 15.2 years (women: 48.9 ± 11.6, men: 61.5 ± 16.4). In this unselected cohort, 12 patients (13%) experienced at least one episode of myasthenic crisis during the 10-year observation period, while 83 patients (87%) were crisis-free. Myasthenic crisis was equally common in the various MG subgroups (Table 1).

**Table 1:** Characteristics of patients with generalized MG who did and did not experience myasthenic crisis.

	**+ Myasthenic crisis (*n* = 12, 12.6%)**	**– Myasthenic crisis (*n* = 83, 87.4%)**	***p*-value**
Age	50.1 ± 14.6	55.1 ± 15.3	0.29
Female	7 (58%) (age: 51.86 ± 13.86)	46 (55%) (age: 48.39 ± 11.9)	0.85
Male	5 (41%) (47.6 ± 21.07)	37 (44%) (63.32 ± 15.06)	
MG onset at age <50 years	8 (66.7%)	49 (59%)	0.76
MG onset at age > 50 years	4 (33.3%)	34 (40.9%)	
MG duration	8.6 ± 7.3 years	11.9 ± 7.2 years	0.14
Onset muscle group: ocular	4 (33%)	40 (48%)	0.393
Onset muscle group: extremity	2 (17%)	10 (12%)	
Onset muscle group: bulbar	6 (50%)	33 (40 %)	
Comorbidities	5 (42%)	54 (65%)	0.21
Anti-AChR ab	10 (83.3%)	60^†^ (75%)	0.736
Anti-MuSK ab	1 (8.3%)	4^†^ (5%)	
No ab detected	1 (8.3%)	16^†^ (20%)	
Normal thymus	5 (41.7%)	35^‡^ (44.9%)	**0.018**
Thymic hyperplasia	0	26^‡^ (32.9%)	
Thymoma	**7 (58.3%)**	17^‡^ (21.5%)	
Previous thymectomy (+)	11 (91.7%)	57 (68.7%)	0.169

^†^The antibody status of three patients in the no-crisis group was unknown; these were not included in the statistics.

^‡^The thymus pathology status of four patients in the no-crisis group was unknown; these were not included in the statistics.

The bold value means that is statistically significant.

Comorbidity categories included malignancies (prostate carcinoma, myelodysplastic syndrome, lymphoma, breast carcinoma, pleomorphic xanthoastrocytoma, adrenal carcinoma, laryngeal carcinoma; 10 patients), autoimmune diseases (polychondritis, familial Mediterranean fever, idiopathic thrombocytopenic purpura, rheumatoid arthritis, Hashimoto's thyroiditis; 10 patients), chronic infections (hepatitis B, tuberculosis; five patients), and others (diabetes mellitus, hypertension, coronary heart disease, chronic obstructive pulmonary disease, stroke, nephrolithiasis, migraine, asthma, osteoporosis; 38 patients). Four patients who experienced myasthenic crisis had hypertension, one had chronic tuberculosis, and one had laryngeal carcinoma. In total, five of the patients in the myasthenic crisis group had a comorbid disease (41%), whereas 54 patients who did not experience a crisis (65%) had a comorbidity. Because of the variation in cancer types, MG subgroups, the time relationship between MG and cancer, and a lack of correlation between the development of MG and the development of malignancies, we do not believe that these were paraneoplastic syndromes. We did not detect any relationship between malignancy treatments and myasthenic crisis.

Thymoma was detected in seven of the 12 patients who experienced a crisis (58%), whereas it appeared in 17 of the 83 patients who did not (21%; *p* = 0.018). Thymic hyperplasia was not diagnosed in any of the patients who experienced myasthenic crisis. Of the 12 patients who experienced a crisis, 11 had undergone a previous thymectomy, seven of them for a thymoma. The only patient who experienced myasthenic crisis and had no previous thymectomy was positive for anti-MuSK antibodies.

Seven patients experienced multiple myasthenic crises, six of them during our 10-year observation period. There was no difference in age and gender between those who experienced a single crisis and those who experienced multiple crises. One patient who experienced multiple myasthenic crises had no detectable muscle antibodies, and one was positive for anti-MuSK antibodies. Five of the seven patients had bulbar muscle onset MG. Four patients had thymoma, and six were thymectomized.

Two patients were first diagnosed with MG during a crisis. One of them had been suffering from diplopia for 2 months and had a mild upper respiratory tract infection immediately before the crisis, whereas the other had rapidly progressive dysphagia and difficulties in breathing. Both were positive for anti-AChR antibodies.

Six patients experienced at least one myasthenic crisis before thymectomy, while six experienced myasthenic crisis only after thymectomy. The time interval from thymectomy to crisis ranged between 3 and 14 years. This time interval did not differ between the patients with and without thymoma (Table 2).

**Table 2:** Timeline of episodes of myasthenic crisis (mc) and clinical characteristics of patients with and without thymoma.

	**Patient**	**Timeline of incidents**		
Thymoma	P1^a^	2014 mc	2014 thymectomy	2021 mc
	P2^a^	2010 thymectomy	2014 mc	2019 mc
	P3^a^	2010 thymectomy	2019 mc	2021 mc
	P4^ab^	2017 mc	2018 mc	2018 thymectomy
	P5^a^	2021 mc	2021 thymectomy	
	P6	2017 thymectomy	2021 mc	
	P7	2011 thymectomy	2014 mc	
No thymoma	P8^c^	2021 mc	2021 mc	No thymectomy
	P9	2021 mc	2021 thymectomy	
	P10	2013 mc	2013 thymectomy	
	P11	2008 thymectomy	2016 mc	2019 mc
	P12^a^	1995 thymectomy	2021 mc	

^a^Bulbar onset MG.

^b^Seronegative MG.

^c^Anti-MuSK MG.

Three episodes of myasthenic crisis occurred during the winter months, with otitis and upper respiratory tract infection as precipitant factors. Two occurred in spring, with a lung abscess and pneumonia as precipitants. Ten episodes of myasthenic crisis occurred during the summer. Five of the ten summer crises occurred in the first 8 years of this 10-year study (0.6 cases per year), while the other five crises coincided with the COVID-19 pandemic during the last 2 years (2.5 cases per year). Of these latter five cases, two of the patients had no symptoms of infection prior to the crisis; one had myositis; one had pneumonia; and one had an upper respiratory infection 3 weeks before the crisis. All of them had negative COVID-19 tests. Finally, three myasthenic crises developed in autumn; two of these were suspected to be triggered by COVID-19 vaccination. One patient experienced escalating dysphagia and generalized weakness 5 days after receiving the BioNTech vaccine; the other developed difficulty in breathing and hoarseness 7 days after receiving the same vaccine. None of our patients developed a myasthenic crisis after COVID-19 infection.

The mean decrease in single breath count from admission to our hospital to the time of ventilatory support was 32% (Table 3). pCO_2_ increased and pO_2_ decreased in parallel; pH values were not affected. HCO3 values were slightly increased. The first parameter to deteriorate was pCO_2_.

**Table 3:** Venous blood gas measurements and reduction in single breath count (SBC) immediately before ventilatory support in 18 cases of myasthenic crisis in 12 patients.

**Total (18 crisis episodes)**	
Decrease in SBC (%)	**31.62** **±12.36**
pH (7.35–7.45)	7.38 ± 0.03
pO_2_ (30–40 mmHg)	**38.61** **±12.76**
pCO_2_ (40–45 mmHg)	**49.27** **±4.75**
$HCO_{3}^{-}$ (24–28 mmol/L)	**27.77** **±2.35**

The bold values mean that they are not in the normal range.

The minimum hospital stay was 5 days, and the maximum was 60 days. The patient with the longest hospital stay experienced recurrent infections with tracheostomy for 34 days. Three patients had complicating urinary tract infections, three had pneumonia, and one developed tonsillitis. Five patients were admitted to the ICU, where they remained for 1, 2, 10, 20 and 55 days there. None of the patients died.

We conducted a PLS analysis to examine the impact of age, gender, decrease in single breath count, time of thymectomy, blood gas condition, thymoma, and presence of comorbidities on length of hospital stay as a measure of severity of myasthenic crisis. No statistically significant correlations were observed (Table 4).

**Table 4:** Impact of MG patient characteristics on length of hospital stay for myasthenic crisis.

	**VIF**	***p*-value**
Age	3.276	0.723
Gender	1.941	0.187
SBC decrease (%)	1.554	0.542
Myasthenic crisis before thymectomy	1.215	0.352
pCO2	2.085	0.158
Thymoma	4.189	0.346
Comorbidities	1.771	0.743

Variance inflation factor (VIF) illustrates multicollinearity: values >3 indicate that the independent variable is highly correlated with one or more of the other variables in a multiple regression equation.

p-value was over the threshold for VIF values in the range 1.215–4.189 (adjusted R^2^: 0.227).

Among the 12 MG patients who experienced a crisis, nine were taking immunosuppressive drugs and 11 were taking acetylcholine esterase inhibitors. Four of them took corticosteroids in doses of 20–80 mg daily, and five took azathiopurine (AZA) at a daily dose of 100 mg. All 18 episodes of myasthenic crisis were treated with IVIG administration. None of the patients had thrombotic events or any renal side effects. Plasmapheresis was given after 2 weeks in the case of three crises, when satisfactory recovery was not achieved with IVIG. In these cases, we preferred to administer plasmapheresis rather than repeat IVIG, taking into account the IVIG half-life and the complete lack of clinical response. Immunosuppressive drug treatment was intensified after 10 of the 18 episodes of myasthenic crisis. The AZA dose was increased to 150 mg daily for three patients, and a new drug was added for seven patients. One patient continued with regular IVIG treatment after the crisis. The dose of acetylcholine inhibitor was also increased after 10 of the episodes of myasthenic crisis. All patients who experienced repeated myasthenic crises were receiving intensified immunosuppressive drug treatment. Rituximab was started in four patients after their second crisis.

## Discussion

In our cohort, we found that thymoma increased the risk of developing a myasthenic crisis, and also increased the risk of multiple crises. Thymectomy did not seem to reduce this risk in thymoma patients. The patients who experienced multiple myasthenic crises were more likely to have bulbar symptom onset. None of our patients who experienced crisis had thymic hyperplasia, so this seems to represent an indicator of low risk. Old age, late-onset MG with AChR antibodies, and MuSK MG may all be associated with a higher risk. MG patients with thymoma tend to have generalized symptoms and more severe clinical forms of the condition (17). Advanced-stage thymoma is a risk factor for post-thymectomy exacerbation, and chemotherapy can induce crisis in MG patients with thymoma (18–20). This was not the case in our patients.

Men were more often affected by generalized MG at older ages, as observed in previous studies (21). During the 10-year period, 13% of all patients with generalized MG developed an episode of myasthenic crisis with the need for ventilatory support. The mean interval from MG onset to crisis was 9 years, which is longer than the intervals reported in recent studies (13, 22). One of our five patients with MuSK antibodies developed a crisis. MuSK MG is generally regarded as a more severe subtype, with a need for effective long-term immunosuppression. All our MuSK patients received such treatment. Some studies have claimed that early-onset MG with AChR antibodies is a risk factor for myasthenic crisis, but we did not find this in our unselected cohort (14, 23). On the contrary, thymic hyperplasia, which is characteristic of this MG subtype, was associated with a decreased risk of myasthenic crisis.

Frequency of comorbidities did not differ between the groups who did and did not experience crisis, nor did comorbidities affect length of hospital stay. Previous studies have obtained conflicting results regarding the relevance of comorbidities in the development and prognosis of myasthenic crisis (23). Diabetes mellitus and pneumonia combined with older age have previously been found to be associated with prolonged hospital stays (22).

We observed an accumulation of myasthenic crises in the summer, in contrast to our expectations, given that more frequent and severe infections occur in autumn and wintertime. High summer temperatures, combined with other precipitating factors that worsen myasthenic complaints, may facilitate the development of myasthenic crisis. Neurotransmission at the neuromuscular junction can be influenced by ambient temperature and weather conditions (24, 25). In addition, summertime crises were more frequent during the period of the COVID-19 pandemic. Asymptomatic COVID-19 infection, false negative COVID-19 test results, and reduced quality of follow-up of MG patients during the period of the pandemic may explain this. Two episodes of myasthenic crises were probably triggered by administration of an mRNA COVID-19 vaccine. This vaccine has previously been reported in single cases to be associated with myasthenic crisis (26, 27). No patients developed a crisis after COVID-19 infection. Our patients who did experience COVID-19 infection generally had mild or moderate disease. Some experienced mild MG exacerbation, but none of these cases progressed to a crisis with the need for ventilatory support. Low viral load, less pathogenic COVID-19 forms, and effective MG treatment may be explanations for this.

In terms of venous blood, pCO_2_ deteriorated before pO_2_ and pH at crisis onset. However, ICU treatment and ventilatory support should not wait until changes in blood gases occur. When forced vital capacity declines to <15 ml/kg or negative inspiratory pressure is <30 cm H_2_O, mechanical ventilation should be started (28, 29). Bilevel positive airway pressure (BiPAP) may be an alternative in patients with milder respiratory weakness (29). Single breath count, together with close and continuous clinical monitoring, is the most important assessment when deciding on ventilatory support, as also illustrated in our cohort. Patients should be observed closely in the hospital during episodes of MG exacerbation with bulbar and respiratory symptoms.

The risk factors for prolonged ICU treatment were infection and age >50 years. A previous study has shown that older age, higher pre-intubation pCO_2_ values, MG-ADL >18, and presence of comorbidities are predictors of prolonged intubation (20). None of our patients died. In the United States, overall in-hospital mortality for MG has been estimated at 2.2%, and for patients with MG crisis, the estimate is 4.7% (9).

The main limitation of our study is the small sample size. However, MG is a rare disease, and myasthenic crises are even rarer. We believe that our single-center approach represents a strength of the study. Because of the retrospective nature of the study, we had to use non-standardized patient assessments, and clinical evaluations were made by different neurologists. Information loss, especially during the pandemic period, represents a weakness of the study.

## Conclusion

Myasthenic crisis continues to represent a risk for patients with MG. Early and effective immunosuppressive treatment of patients with generalized MG and prevention of potentially precipitating events, such as infections, reduce the risk of myasthenic crisis. This is especially important in high-risk MG patients: those with a thymoma and those who have previously experienced myasthenic crisis. Among our patients who experienced myasthenic crisis, none had thymic hyperplasia; this seems to be a risk-reducing factor. Bulbar onset of MG represents a risk factor for multiple myasthenic crises. Early access to intensive care with active and individualized immunosuppression, combined with treatment of complications, provides a good outcome for myasthenic crisis.

## Data availability statement

The original contributions presented in the study are included in the article/supplementary material, further inquiries can be directed to the corresponding author.

## Ethics statement

The studies involving human participants were reviewed and approved by the Haydarpasa Numune Hospital Scientific Ethical Committee. Written informed consent for participation was not required for this study in accordance with the national legislation and the institutional requirements.

## Author contributions

Concept, design, analysis and/or interpretation, control, and revisions: SO, NG, and KT. Processing: SO and EN. Literature search: SO, EN, KT, and NG. Writing: SO. Supervising: KT and EN. All authors contributed to the article and approved the submitted version.
